# Continuation of breastfeeding after surgical removal of AQUALIFT^Ⓡ^ due to mastitis: A case report

**DOI:** 10.1016/j.jpra.2025.08.007

**Published:** 2025-08-06

**Authors:** Kayoko Uno, Yushi Suzuki, Yukie Nakamura, Kazuo Kishi

**Affiliations:** a Department of Plastic and Reconstructive Surgery, Ofuna Chuo Hospital, 6-2-24 Ofuna, Kanagawa Kamakura 247-0056, Japan; b Department of Plastic and Reconstructive Surgery, Keio University School of Medicine, 35 Shinanomachi, Tokyo Shinjuku 160-8582, Japan

**Keywords:** AQUALIFT^Ⓡ^, Breast augmentation, Breastfeeding, Copolyamide, Surgical removal

## Abstract

Several complications have been reported following copolyamide filler injections for breast augmentation leading many countries to issue warnings regarding the use of nonabsorbable fillers. While previous reports have described breast complications necessitating surgical intervention after filler injections, none have explicitly documented the continuation of postoperative breastfeeding. Here, we report a rare case in which a woman with a history of breast augmentation using AQUALIFT^Ⓡ^ developed mastitis following childbirth and the initiation of breastfeeding. Despite requiring surgical removal of the AQUALIFT^Ⓡ^, she successfully continued breastfeeding owing to a strong desire and commitment to regular milk expression throughout the perioperative period.

Nevertheless, the carcinogenicity and toxicity of copolyamide, its presence in breast milk, and its effects on breastfed infants remain unclear. While various authorities have issued warnings concerning the use of fillers for breast augmentation, a significant number of women have already undergone this procedure. As these women reach childbearing age, further research is required to evaluate the safety of breastfeeding after filler-based augmentation.

## Introduction

Complications associated with breast augmentation using copolyamide fillers have recently been reported. Since 2016, multiple countries have issued warnings against the use of nonabsorbable fillers for breast augmentation.^1^ However, no documented cases concerning patients continuing breastfeeding after surgical intervention for complications related to copolyamide fillers have been reported. Here, we present a case of a patient who, despite requiring surgical removal of the AQUALIFT^Ⓡ^, a type of copolyamide filler, owing to mastitis, was able to continue breastfeeding driven by a strong desire to do so.

## Case presentation

**Figure 1:**
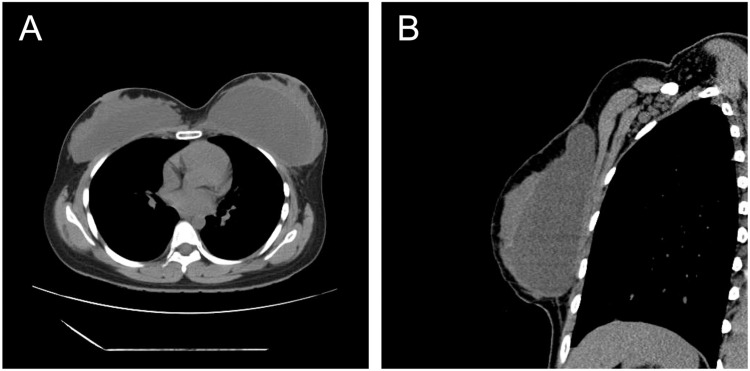
Computed Tomography (CT) image obtained prior to surgical removal. (A): Axial CT image. The left breast with the copolyamide filler appears swollen. (B): Sagittal CT image. The copolyamide filler is localized between the posterior mammary gland and the anterior pectoralis major muscle.

**Figure 2:**
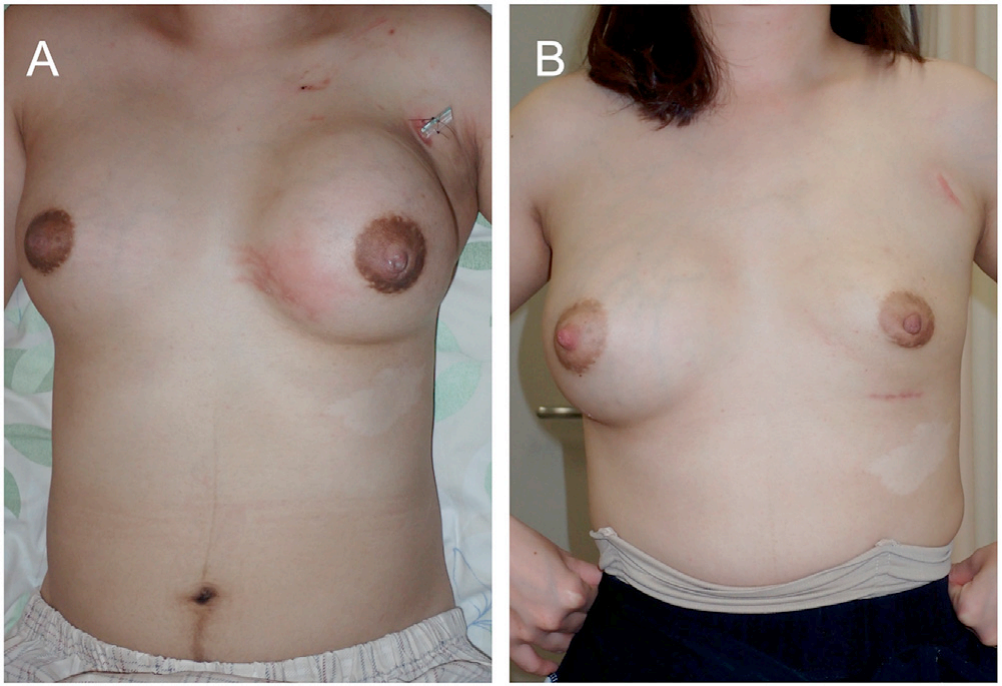
Clinical presentation of the patient. (A): Immediately after rupture in the supine position. The left breast was swollen compared with the right breast, with an enlarged erythematous area extending inferiorly. (**B):** Six months following surgical removal in the standing position. The left breast volume has decreased.

**Figure 3:**
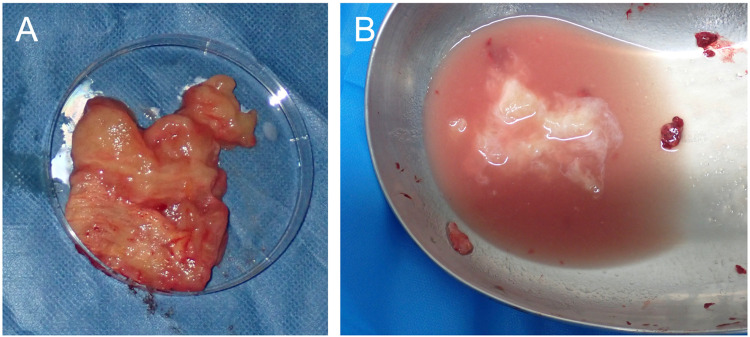
Copolyamide filler condition. (A): Filler immediately following removal. It retained its solid, pale-yellow, jelly like form, mixed with cloudy exudate. (B): Filler after agitation in a saline solution. The mixture transformed into a milky liquid.

A 29-year-old woman underwent bilateral breast augmentation using AQUALIFT^Ⓡ^ approximately 7 years prior at another cosmetic clinic. After childbirth and initiation of breastfeeding, she developed redness and swelling in her left breast. As her symptoms persisted, she was referred to our department, presenting with swelling and fever, and erythema in the superior portion of her left breast, corresponding to the AQUALIFT^Ⓡ^ injection site. Computed tomography (CT) findings identified foreign material located between the posterior mammary gland and the anterior pectoralis major muscle (Figure 1). She was diagnosed with cellulitis and mastitis. Although she had already been prescribed cefcapene pivoxil hydrochloride hydrate 100 mg orally three times daily for 3 days at a previous clinic, her symptoms did not improve. Given her strong desire to continue breastfeeding, she was treated with cefaclor 500 mg orally three times daily for 16 days at our department. However, her symptoms worsened with increasing swelling and skin tension. Eventually, the erythematous area ruptured, discharging white exudate (Figure 2). Owing to persistent symptoms and a high risk of infection in the AQUALIFT^Ⓡ^ region, surgical removal of the filler was performed under general anesthesia. Despite compression of the affected breast, only a small amount of pale-yellow, jelly like material mixed with cloudy exudate was discharged from the rupture site, making aspiration-based removal challenging. We removed a substantial amount of cloudy fluid and jelly like foreign material through a 5 cm skin incision along the inframammary fold. The area was then irrigated with saline. The extracted filler maintained its solid form when removed, but transformed into a milky liquid when agitated in saline (Figure 3). Given her strong reluctance to discontinue breastfeeding, she continued to express milk from her right breast during hospitalization and resumed on the affected side on postoperative day 1. She received cefazolin 1 g intravenously every 8 h for 1 day during the perioperative period, and no further signs of infection were observed thereafter. Six months postoperatively, she was continuing to breastfeed without any recurrence in the left breast nor development of new symptoms in the right breast. The wound at the rupture site developed into a hypertrophic scar, which was treated with triamcinolone acetonide. In contrast, the surgical incision made through the inframammary fold healed without complications. The infant did not experience any adverse effects.

## Discussion

Saline and silicone implants as well as fat transfer are well-established methods for breast augmentation. Dermal filler-based breast augmentation has historically been considered a simpler and less invasive alternative. Since 1900, various substances such as paraffin, vegetable oils, lanolin, beeswax, and silicone gel had been used as dermal fillers. However, severe complications including pulmonary embolism, ulceration, inflammation, induration, erythema, granuloma formation, and material migration, have resulted in their use being widely banned.^2^

In the late 1990s, polyacrylamide hydrogel (PAAG) was developed as a new nonabsorbable filler and gained popularity owing to its convenience. However, PAAG migration, infection, mass formation, and other intractable conditions led to its ban in China in 2006.^3^ In 2005, AQUAFILLING^Ⓡ^ (Biomedica, Spol. s. r. o., Czech Republic) was developed as a copolyamide filler distinct from PAAG. It was initially approved by the Korean Food and Drug Administration for limited applications such as facial use.^4^ In 2015, Shin et al. reported on breast augmentation using AQUAFILLING^Ⓡ^ with implants,^5^ leading to an increase in its use as a high-volume filler for breast augmentation. However, numerous complications associated with AQUAFILLING^Ⓡ^ soon emerged, prompting the Korean Society of Aesthetic Reconstructive Breast Surgery to issue a warning against its use for breast augmentation in 2016.^1^ More recently, in 2022, the Italian Society of Aesthetic Reconstructive Breast Surgery also issued a warning against AQUAFILLING^Ⓡ^.^1^

During this period, AQUAFILLING^Ⓡ^ was rebranded as LOS DELINE^Ⓡ^ (BIOTRH, s. r. o., Czech Republic) in 2018.^1^ AQUALIFT^Ⓡ^ (National Medical Technologies Center Co., Ltd., Ukraine), the filler used in our reported case, is another copolyamide-based dermal filler that was marketed around the same time and subsequently renamed as ACTIVEGEL^Ⓡ^ (National Medical Technologies Center Co., Ltd., Ukraine) in 2015.^1^

In 2021, Nomoto et al. examined and analyzed copolyamide fillers, PAAG, and two types of PAAG fillers. Their study findings indicated that copolyamide fillers may leave resin components in the body and that copolyamide fillers closely resemble PAAG and PAAG fillers in composition. These findings suggest that the risks associated with copolyamide fillers should be considered equivalent to those of PAAG fillers.^6^

Research has described complications following breast augmentation with copolyamide or PAAG fillers, particularly after the initiation of breastfeeding, often necessitating surgical removal.^1^ However, no research has explicitly stated whether the patients could continue breastfeeding postoperatively. In our case, the foreign material was localized, encapsulated in a fibrous capsule. This anatomical positioning may have minimized damage to the mammary glands and lactiferous ducts, potentially preserving the lactation function. Nevertheless, the effects of copolyamide and PAAG fillers on breastfeeding remain uncertain as their carcinogenicity, toxicity, and possible presence in breast milk have not been fully investigated.^7^

While the precise number of patients who have undergone breast augmentation with copolyamide fillers is unknown, PAAG fillers were used in approximately 200,000 patients in China over a decade prior to their prohibition.^3^ Given the compositional and procedural similarity between PAAG and copolyamide fillers, it is plausible that copolyamide fillers may have been used in a comparable number of cases, but further research is needed to verify this assumption.

A review of copolyamide-related cases reported symptom onset ranging from 1 to 60 months postoperatively.^1^ However, in one reported case of PAAG filler breast augmentation, complications were reported 15 years postoperatively, triggered by breastfeeding.^8^ Given the structural similarity between PAAG and copolyamide fillers, delayed complications may occur over even longer periods than currently reported. Moreover, as more women undergo childbirth at an older age, cases of long-dormant filler-related complications triggered through breastfeeding may become more frequent.

Breastfeeding is often recommended,^9^ reinforcing the widespread belief that it is inherently beneficial and desirable. This societal expectation extends to women who have undergone breast augmentation with dermal fillers. However, owing to the lack of conclusive evidence regarding the safety of breastfeeding after filler-based augmentation, it remains uncertain whether breastfeeding should be contraindicated, whether fillers should be removed beforehand, or whether breastfeeding can be permitted despite unknown safety risks, as long as lactating women acknowledge this uncertainty.

In our case, the patient continued breastfeeding from both the unaffected and affected sides, with no observed short-term impairments in terms of feeding the baby. Further research is necessary to evaluate the long-term safety of copolyamide fillers and the potential presence of copolyamide and PAAG degradation products in breast milk.

## Conclusion

Here, we report a case in which lactation was preserved by continuing milk expression following surgical filler removal. Given that many women have undergone dermal filler-based breast augmentation prior to childbirth and lactation, further studies are required to assess the effects of copolyamide and PAAG fillers on breast milk. In addition, increasing awareness of this issue among obstetricians and breast surgeons is essential.

## Informed consent

The patient whose case is presented has provided informed consent for the publication of the manuscript.
